# Access to Care and Self-Rated Health Status: Comparison of Rural and Urban Immigrants in the US

**DOI:** 10.1007/s10903-025-01846-z

**Published:** 2026-01-13

**Authors:** Redwan Bin Abdul Baten, Kiran Thapa, Altaf Saadi

**Affiliations:** 1 University of North Carolina at Charlotte, Charlotte, USA; 2 Harvard University, Cambridge, USA; 3 Massachusetts General Hospital, Boston, USA

**Keywords:** Rural, Immigrant, Disparity, Access to care, Health status

## Abstract

Existing immigrant health literature has highlighted disparities among US immigrants but focused primarily on urban immigrants. To assess utilization patterns and differences in access to care, healthcare utilization, and health status between rural and urban immigrants. We conduct a cross-sectional secondary analysis using the National Health Interview Survey (NHIS) data from 2019-2023. Multivariable Ordinary Least Squares (OLS) regression models estimate the differences in immigrant health by their rural and urban location. 18-64-year-old foreign-born adults residing in the US rural or urban areas were included. Access to care indicators include health insurance, having a usual place of care, being unable to pay medical bills, and delayed filling prescriptions to save money. Healthcare utilization indicators include being in a hospital overnight, the number of urgent care or ER/ED visits, and whether the last visit was a wellness visit. Self-reported health measures the respondents' health status. Compared to urban, rural immigrants are significantly less likely to have private health insurance coverage (4 percentage points or pp) and urgent care visits (9 pp); and significantly more likely to have - usual sources of care (3 pp), problems paying medical bills (2 pp), delay in filling prescriptions (3 pp), emergency room/department visits (4 pp), and poor health (2 pp). This study highlights disparities in access to care and health outcomes among rural and urban immigrants. Policymakers should consider that the unique challenges faced by rural immigrants are further intensified by the difficulties of rural life in the US.

**Supplementary Information:**

The online version contains supplementary material available at 10.1007/s10903-025-01846-z.

## Introduction

The United States (US) is home to over 45 million immigrants, representing 14% of the US population [1]. Although most immigrants tend to live in or around major cities, recent decades have seen a shift, with more settling in rural areas [2, 3]. Between 2000 and 2018, immigrants accounted for 37% of overall population growth in rural counties, mirroring their growth in urban counties [4]. While the population of US-born residents in most rural counties declined, rural immigrants tend to be low-wage, unskilled workers without college degrees, and are less likely to be naturalized citizens or have health insurance [5]. While existing literature has highlighted disparities among US immigrants, it has focused primarily on urban immigrants [6–8]. Focusing exclusively on urban immigrants’ healthcare needs overlooks the unmet healthcare needs, disparities, and challenges of rural immigrants who are at the intersection of these communities.

Rural residents have lower life expectancy, poorer health status, and less access to quality healthcare services than urban residents [9, 10]. Existing disparities in rural healthcare and mortality are further exacerbated by increasing hospital and nursing home closures in rural communities [11, 12]. While immigration status is a major driver of unequal healthcare coverage throughout the US, immigrants in rural areas face additional challenges such as lower education, high poverty, limited broadband access, language access barriers or interpretation services, and transportation and distance barriers that their living in rural areas may compound [13–17]. These may disproportionately affect immigrants who are Black or Hispanic, have limited English proficiency, have chronic conditions/disabilities, or are undocumented. Most existing studies examining the experiences of rural immigrants are qualitative, focus on a specific ethnic group, or are limited to a particular city or region [18, 19]. 

We fill this gap by using five years of data from the National Health Interview Survey (NHIS 2019–2023) to assess utilization patterns and differences in access to care and self-reported health status between rural and urban immigrants.

## Methods

### Study Design and Data

We conduct a cross-sectional secondary analysis using the NHIS, a representative US population survey conducted annually among approximately 45,000 households with around 100,000 people residing. We used the harmonized NHIS 2019–2023 data from the Integrated Public Use Microdata Series (IPUMS) and included 18-64-year-old foreign-born adults residing in US rural or urban areas. Due to the differential healthcare needs of these age groups and variation in access to health insurance by citizenship status and state Medicaid eligibility criteria for immigrants, a smaller sample size, missing data on key covariates (such as labor force participation, education, marital status etc.), we exclude from the analysis individuals aged 1–17 years and those aged 65 years and above. The study was deemed exempt by the Institutional Review Board (IRB) of the University of North Carolina at Charlotte because it used publicly available data.

### Outcomes

We include several indicators of healthcare access and health status as outcomes. Health insurance indicators include – (i) having any health insurance (0/1), (ii) having private health insurance (0/1), and (iii) having health insurance through Medicaid (0/1). Access to care binary indicators include: (i) having a usual place of care in the past 12 months, (ii) being unable to pay medical bills, and (iii) delayed filling prescriptions to save money. Last year’s healthcare utilization indicators include: (i) being in a hospital overnight, (ii) number of times visited urgent care (continuous), (iii) number of times visited ER/ED (continuous), and (iv) whether the last visit was a wellness visit. We include two measures of health status: (i) self-rated health in five categories from 1 (poor) to 5 (excellent), and (ii) a binary indicator for good/very good/excellent health.

### Key Independent Variable

Our key independent variable is the respondents’ rurality. The NHIS dataset includes a four-category indicator for rural-urban location, including non-metropolitan, sizable central metro, large fringe metro, medium and small metro counties. The 2013 National Center for Health Statistics (NCHS) Classification Scheme for Counties classifies counties into four levels based on respondents’ locations [20]. We use the NCHS classification to categorize non-metropolitan counties as rural and sizable central metro, large fringe metro, and medium and small metro counties as urban.

### Covariates

Following previous literature, the analytic models control for demographic characteristics [21, 22]. Demographic characteristics included in the analytic models were age (18–34/35–49/50–64), sex (male/female), race (White/Black/Asian/Others), education (less than high school/high school/college/postgraduate), marital status (single/separated or divorced or widowed/married), language is spoken at home (English/not English), labor force participation (yes/no), citizenship (yes/no), years in the US (< 1/1–5/5–10/10–15/>15), poverty status (< 100% FPL, 100–149% FPL, 149–400% FPL, > 400% FPL), and region of residence (Northeast//Midwest/South/West). NHIS reports the US region in which the housing unit containing the survey participants was located. The four regions - Northeast, North Central/Midwest, South, and West correspond to the U.S. regions recognized by the Census Bureau.

### Statistical Analysis

Descriptive statistics, including weighted percentages and chi-square tests, are performed to analyze differences in the study variables between urban and rural immigrants. Multivariable Ordinary Least Squares (OLS) regression models were employed to estimate the differences between rural and urban immigrants’ health insurance, access, healthcare utilization, and health status. We utilize OLS models as the coefficients derived from linear regressions are directly interpretable and yield unbiased estimates of treatment effects on binary outcomes. The main model is specified as follows:

1 $$ {\mathrm{Y}}_{{{\mathrm{iat}}}} = {\text{ }}\beta _{0} + {\text{ }}\beta _{{\mathrm{1}}} {\text{ Rural}}_{{\mathrm{a}}} + {\text{ }}\beta _{{\mathrm{2}}} {\text{ X}}_{{{\mathrm{iat}}}} + {\text{ }}\theta _{{\mathrm{t}}} + {\text{ }}\alpha _{{\mathrm{a}}} + {\text{ }}\varepsilon _{{{\mathrm{iat}}}}$$

In this model, Y is one of the outcomes for individual i in rural/urban areas a in year t. Rural_a_ is the rural-urban location of the respondent; X_iat_ includes individual-level covariates; θ_t_ is year-fixed effects, which capture national trends in the study outcomes shared across rural and urban areas; α_a_ includes regional fixed effects, which account for time-invariant differences. In the model, β_1_ estimates the difference in outcome between rural and urban immigrants.

To generalize the results across all areas, robust standard errors are clustered at the respondents’ region level. The analytic models are weighted by the IPUMS NHIS weights provided with the dataset. The alpha level was set at 0.10, and all analyses are performed using Stata 17.0.

## Results

### Sample Characteristics

The full sample included 21,061 respondents, where 20,462 were living in urban areas, and 1,139 were living in rural areas. Only 4.15% of the immigrant study sample lived in rural areas. Rural immigrants were older, more likely to identify as White or Other as their racial identity, more likely to be of white race and Hispanic ethnicity, more likely to speak English, more likely to live in the Midwest and South regions of the US, have lower educational attainment, and less likely to be married (see Table 1).

**Table 1:** Descriptive statistics of the study analytical sample of non-US-born 18-64-year-old Immigrants, 2019–2023 NHIS

Variables	Weighted Percent or Mean			
	Full Sample Unweighted *N* = 21,061 Weighted *N* = 21,601	Urban Sample Unweighted *N* = 20,462 Weighted *N* = 20,503	Rural Sample Unweighted *N* = 1,139 Weighted *N* = 1,098	*P*-value
***Age***				
18–34%	27.88 (0.00)	27.92 (0.00)	25.54 (0.02)	0.00
35–49%	39.50 (0.00)	39.55 (0.00)	39.54 (0.02)	
50–64%	32.62 (0.00)	32.53 (0.00)	34.93 (0.02)	
***Sex***				
Male %	48.20 (0.01)	48.27 (0.01)	44.74 (0.02)	0.59
Female %	51.80 (0.01)	51.73 (0.01)	55.26 (0.02)	
***Race***				
White %	40.88 (0.00)	40.70 (0.01)	48.09 (0.02)	0.00
African American %	10.31 (0.00)	10.47 (0.00)	5.11 (0.01)	
Asian %	25.10 (0.00)	25.61 (0.00)	12.55 (0.01)	
Others %	23.71 (0.00)	23.21 (0.00)	34.24 (0.02)	
***Ethnicity***				
Not Hispanic %	51.72 (0.01)	52.39 (0.01)	36.76 (0.02)	0.04
Hispanic %	48.28 (0.01)	47.63 (0.01)	63.23 (0.02)	
***Education***				
Less than High school %	22.18 (0.00)	21.58 (0.00)	34.61 (0.02)	0.00
High school graduate %	24.10 (0.00)	23.83 (0.00)	29.12 (0.02)	
College graduate %	39.19 (0.00)	39.60 (0.01)	30.58 (0.02)	
Postgraduate %	14.53 (0.00)	15.00 (0.00)	5.68 (0.01)	
***Marital Status***				
Single %	26.46 (0.00)	26.43 (0.00)	25.55 (0.02)	0.30
Separated/ Divorced/ Widowed %	10.20 (0.00)	10.09 (0.00)	12.36 (0.01)	
Married %	63.34 (0.00)	63.48 (0.01)	62.08 (0.02)	
***Language Spoken***				
Speaks English %	76.33 (0.00)	76.22 (0.00)	79.77 (0.02)	0.00
Does not Speak English %	23.67 (0.00)	23.78 (0.00)	20.23 (0.02)	
***Labor Force Participation***				
Not in Labor Force %	24.51 (0.00)	24.37 (0.00)	27.23 (0.02)	0.00
In Labor Force %	75.49 (0.00)	75.63 (0.00)	72.77 (0.02)	
***Citizenship***				
No %	49.51 (0.01)	49.05 (0.01)	60.34 (0.02)	0.00
Yes %	50.49 (0.01)	50.95 (0.01)	39.66 (0.02)	
***Years in the US***				
< 1 year %	1.18 (0.00)	1.16 (0.00)	1.59 (0.01)	0.00
1–5 years %	8.97 (0.00)	9.08 (0.00)	6.62 (0.01)	
5–10 years %	12.49 (0.00)	12.57 (0.00)	9.70 (0.01)	
10–15 years %	11.11 (0.00)	11.18 (0.00)	9.73 (0.01)	
15 + years %	66.25 (0.00)	66.01 (0.00)	72.36 (0.02)	
***Poverty Category***				
<100 FPL %	14.50 (0.00)	14.18 (0.00)	21.97 (0.02)	0.00
>=100 & <=149 FPL %	13.36 (0.00)	13.31 (0.00)	14.45 (0.02)	
>=149 & <=400 FPL %	39.26 (0.01)	39.00 (0.00)	45.36 (0.02)	
>400 FPL %	32.87 (0.00)	33.51 (0.00)	18.23 (0.02)	
***Region***				
Northeast %	19.62 (0.00)	20.10 (0.00)	9.39 (0.01)	0.00
Midwest %	11.43 (0.00)	10.98 (0.00)	21.57 (0.02)	
South %	35.89 (0.00)	35.62 (0.01)	42.58 (0.02)	
West %	33.06 (0.00)	33.30 (0.00)	26.46 (0.02)	
***Location***				
Urban %	95.85 (0.00)			
Rural %	4.15 (0.00)			
**Outcome Variables**				
***Health Insurance***				
Any health insurance coverage %	76.01 (0.00)	76.70 (0.00)	59.90 (0.02)	0.00
Private health insurance coverage %	58.37 (0.01)	59.13 (0.01)	44.57 (0.02)	0.00
Health insurance through Medicaid %	12.32 (0.00)	12.31 (0.00)	9.88 (0.01)	0.39
***Access to Care***				
Has usual place for medical care %	84.28 (0.00)	84.28 (0.00)	85.19 (0.02)	0.08
Problems paying or unable to pay medical bills past 12 months %	11.29 (0.00)	10.99 (0.00)	16.56 (0.01)	0.00
Delayed filling prescription to save money, past 12 months %	3.55 (0.00)	3.45 (0.00)	6.04 (0.01)	0.00
***Healthcare Utilization***				
Was in a hospital overnight in past 12 months %	5.85 (0.00)	5.77 (0.00)	7.40 (0.01)	0.00
Number times visited urgent care in past 12 months (mean)	0.42 (0.01)	0.42 (0.01)	0.35 (0.04)	0.33
Number times in ER/ED in past 12 months (mean)	0.23 (0.01)	0.23 (0.01)	0.29 (0.03)	0.00
Last doctor visit was a wellness visit %	81.90 (0.00)	82.05 (0.00)	78.25 (0.02)	0.00
***Self-Reported Health Status***				
Five Categories (mean)	3.75 (0.01)	3.76 (0.01)	3.54 (0.05)	0.00
Good/Very Good/Excellent %	88.90 (0.00)	89.08 (0.00)	84.77 (0.02)	0.00

Analysis of NHIS data 2019–2023. The descriptive statistics of the variables are based on the regression estimation sample for health insurance as the dependent variable. P-values obtained from chi-square tests. NHIS: National Health Interview Survey. FPL: Federal Poverty Level

59.90% of rural immigrants had health insurance compared to 76.70% of urban immigrants (χ^2^ *p* < 0.001). Health insurance through private coverage (44.57% vs. 59.13%; χ^2^ *p* < 0.001) was lower for rural immigrants than their urban counterparts. A higher percentage of rural immigrants reported having a usual place for medical care (85.19% vs. 84.28%; χ^2^ *p* = 0.08)), being unable to pay medical bills (16.56% vs. 10.99%; χ^2^ *p* < 0.001), and having delayed filling prescriptions to save money (6.04% vs. 3.45%; χ^2^ *p* < 0.001) in the past 12 months. Rural immigrants had a higher percentage of being in a hospital overnight (7.40% vs. 5.77%; χ^2^ *p* < 0.001) and a lower percentage of the last doctor visit being a wellness visit (78.25% vs. 82.05%; χ^2^ *p* < 0.001). Rural immigrants utilized emergency care more often than urban immigrants (χ^2^ *p* < 0.001). Rural immigrants were also less likely to report good, very good, or excellent health than urban immigrants (84.77% vs. 89.08%) (Table 1).

### Association with Insurance coverage, Access to Sare, and Healthcare Utilization

We observed disparities between rural and urban immigrants concerning insurance coverage types, access to care, and utilization measures (Table 2). Rural immigrants are 4% points (pp) (*p* < 0.10, s.e. 0.01) or 6.8% less likely to have private insurance coverage. Similarly, rural immigrants have a three pp (*p* < 0.10, s.e. 0.01) or 3.6% higher likelihood than urban respondents to have a usual place for medical care. Rural immigrants have a three pp (*p* < 0.05, s.e. 0.01) or 27.3% higher likelihood of being unable to pay medical bills and two pp (*p* < 0.05, s.e. 0.00) or 58.0% higher likelihood of delaying filling prescriptions to save money than urban immigrants. Rural immigrants have a four pp higher likelihood of having emergency room visits (*p* < 0.10, s.e. 0.02) or 17.4% and a nine pp lower likelihood of having urgent care visits (*p* < 0.05, s.e. 0.01) or 21.4% in the past 12 months than urban immigrants.

**Table 2:** Differences in health insurance, access to care, healthcare utilization, and health status between rural and urban non-US-born 18-64-year-old Immigrants, 2019–2023 NHIS

Outcome Variables	Coefficient (S.E.)
***Health Insurance***	
Any health insurance coverage	−0.07 (0.04)
Private health insurance coverage	−0.04* (0.01)
Health insurance through Medicaid	−0.03 (0.02)
***Access to Care***	
Has usual place for medical care	0.03* (0.01)
Problems paying or unable to pay medical bills, past 12 months	0.03** (0.01)
Delayed filling prescription to save money, past 12 months	0.02** (0.00)
***Healthcare Utilization***	
Was in a hospital overnight in past 12 months	0.01 (0.01)
Number times visited urgent care in past 12 months	−0.09** (0.02)
Number times in ER/ED in past 12 months	0.04* (0.02)
Last doctor visit was a wellness visit	−0.02 (0.03)
***Self-Reported Health Status***	
Five Categories (5 = Excellent, 1 = Poor)	−0.11** (0.02)
Binary (Good/Very Good/Excellent vs. Poor/Fair)	−0.02* (0.01)

Standard errors are in parentheses and clustered by the respondent’s region. Analysis of NHIS data 2019–2023 using Ordinary Least Square (OLS) models. Estimates are weighted by NHIS sampling weights and adjusted for the following demographic factors - age, sex, race, education, marital status, citizenship, language, region of residence, labor force participation, poverty status, years in the US, and year and region fixed effects. Sample size ranges from 17,031 to 17,071. * *p* < 0.10, ** *p* < 0.05, *** *p* < 0.01. NHIS: National Health Interview Survey. S.E.: Standard error

### Association with Health Status

There is a significant difference in self-rated health (0.11 points on the 1–5 points scale) for rural immigrants compared to urban immigrants (*p* < 0.50, s.e. 0.02). Rural immigrants have a two pp (*p* < 0.10, s.e. 0.01) or 2.2% lower likelihood of reporting good/very good/excellent health than poor /fair health compared to urban immigrants (Table 2).

### Robustness Checks

Several robustness checks are performed to determine the sensitivity of our results. In one test, we exclude 2019–2020 because of substantial disruptions in health care access during the COVID-19 pandemic (Supplemental Table 1). The results from the robustness checks are generally similar to those from the main analyses for insurance coverage, access to care, healthcare utilization, and health status. In another check, we analyze those aged 65 and above (Supplemental Table 2). We observe that the coefficients differ in magnitude and direction from those for 18-64-year-olds. This demonstrates the divergent health status and access to care for those aged 65 and above. The results confirm the validity of our study estimates.

## Discussion

Our study utilizes a nationally representative US dataset to reveal disparities in healthcare use and outcomes among rural and urban immigrants. Compared to immigrants in urban counties, immigrants in rural counties were significantly less likely to have private health insurance coverage, more likely to have usual source of care, more likely to have problems paying or unable to pay medical bills, more likely to delay filling prescription to save money, more likely to have emergency room/department visits, less likely to have urgent care visits, and more likely to report poor health. These findings reflect the existing rural-urban disparities in access to care in the US but add to the literature by highlighting the challenges immigrants face in rural communities that have not yet been fully elucidated.

The difference in insurance coverage reflects existing rural-urban and immigrant disparities. Rural residents are more likely to have public or no coverage and less likely to have private coverage [23, 24]. In 2022, only 54% of immigrants had private health insurance compared to 68% of US-born individuals. Consistent with this, we found that rural immigrants were also more likely to have lower private healthcare insurance. However, we did not find significant differences in access to any healthcare coverage or Medicaid. Lower private coverage could be because immigrants living in rural areas work in low-paying jobs that are less likely to provide employer-sponsored coverage [25]. Restrictions such as the ‘five-year bar’ for lawfully present immigrants leave many immigrants ineligible for insurance subsidies [26]. 

Rural immigrants reporting a higher usual source of care in our study is also consistent with findings among rural residents [27]. Despite reporting a higher usual source of care, research has shown that rural residents report fewer visits to specialists and travel longer to see providers who are less likely to be physicians [27–33]. They are also more likely to struggle to pay medical bills or delay medical care due to cost [27, 29]. We find that rural immigrants reported more significant problems in paying medical bills or filling prescriptions. This could be related to the lack of insurance coverage, which often translates to lower preventative and specialty care service utilization, delayed or forgone care, and high out-of-pocket medical expenditures and debt [24, 34]. We also find that ED visits were significantly higher among rural than urban immigrants. Rural residents visit emergency departments at higher rates than urban residents for urgent and non-urgent care [35, 36]. Urgent Care Centers (UCC) mainly serve urban or suburban areas with high proportions of privately insured patients [37], and only about 9% of over 14,900 UCCs were in rural areas in 2024 [38]. This potentially explains our findings of lower UCC visits by rural immigrants.

We find that rural immigrants reported significantly worse self-reported health than urban immigrants. Rural residents report disproportionately higher rates of poor health behaviors, chronic conditions, disabilities, and mortality [39, 40]. Immigrants are postulated to have a health advantage over US citizens that diminishes over longer residency [41]. It is not clear whether this process is accelerated in rural versus urban settings.

Policy solutions include greater health insurance coverage, including for immigrants [42, 43]. The Affordable Care Act (ACA) has made it easier for rural residents and immigrants to access healthcare [43, 44]. Medicaid expansion under the ACA is linked to declines in trouble-paying medical bills, reductions in medical debt, decreased emergency department visits, decreased catastrophic health expenditures, and greater financial performance of rural hospitals [45–47]. Immigrants with poor English proficiency may be particularly vulnerable due to the limited availability of rural resources for language translation and interpretation services [48]. Research has shown that immigration may help fill critical shortages of healthcare professionals and increase access to care in rural areas [49], and boosts economic growth while reducing healthcare access disparities [49, 50]. Therefore, solutions that meet rural immigrant needs can have broader community-level benefits.

Our study’s limitations include the inability to distinguish differences in immigration status, lack of granularity about the usual source of care, inability to distinguish between different aspects of care, and inability to study potential disparities in mental health and substance use disorder treatment services. Another limitation is the small sample size of immigrants living in rural areas. We did not compare the sample of immigrants to the US-born population, which limits our ability to make inferences about the overall US population. Finally, our rural immigrant sample was more likely to be Hispanic ethnicity and less likely to be Black/African or Asian race. There may be important nuances regarding how race and ethnicity intersect with immigration and rurality that future studies could explore. For example, prior studies have shown synergistic health disadvantage among Black/African individuals living in rural areas, suggesting that structural racism and disinvestment in rural communities may compound health harms for these groups. Future research should examine these nuances in greater depth [51, 52].

## Conclusions

There are marked disparities in access to care and health outcomes among rural and urban immigrants. Efforts to mitigate health disparities must consider the plight of rural immigrants, whose unique challenges are compounded by the difficulties of US rurality. Revitalizing rural healthcare requires a multi-sectoral collaboration that prioritizes equitable resource distribution, financial sustainability, and infrastructural support for rural counties, while also considering the growing immigrant population.

## Supplementary Information

Below is the link to the electronic supplementary material.

Supplementary Material 1

## Data Availability

Data will be provided upon request.
