# Supplementary material for: Access to Care and Self-Rated Health Status: Comparison of Rural and Urban Immigrants in the US
Source: J Immigr Minor Health. 2026 Jan 13;28(4):945–52. doi: 10.1007/s10903-025-01846-z (PMC13451389; doi:10.1007/s10903-025-01846-z)
Supplement: Supplementary file 1 [file 10903_2025_1846_MOESM1_ESM.docx]

**Supplemental Table 1:** Differences in health insurance, access to care, healthcare utilization, and health status between rural and urban non-US-born 18-64-year-old Immigrants, 2019-2023 NHIS.

| **Outcome Variables** | **Coefficient (S.E.)**  **2019-2023** | **Coefficient (S.E.)**  **2021-2023** |
| --- | --- | --- |
| **Health Insurance** | |  |
| Any health insurance coverage | -0.07 (0.04) | -0.07 (0.05) |
| Private health insurance coverage | **-0.04* (0.01)** | **-0.02* (0.01)** |
| Health insurance through Medicaid | -0.03 (0.02) | -0.04 (0.03) |
| **Access to Care** | |  |
| Has usual place for medical care | **0.03* (0.01)** | 0.03 (0.02) |
| Problems paying or unable to pay medical bills, past 12 months | **0.03** (0.01)** | 0.02 (0.01) |
| Delayed filling prescription to save money, past 12 months | **0.02** (0.00)** | **0.02** (0.01)** |
| **Healthcare Utilization** | |  |
| Was in a hospital overnight in past 12 months | 0.01 (0.01) | 0.01 (0.01) |
| Number times visited urgent care in past 12 months | **-0.09** (0.02)** | **-0.14** (0.04)** |
| Number times in ER/ED in past 12 months | **0.04* (0.02)** | 0.04 (0.03) |
| Last doctor visit was a wellness visit | -0.02 (0.03) | -0.00 (0.02) |
| **Self-Reported Health Status** | |  |
| Five Categories (5=Excellent, 1=Poor) | **-0.11** (0.02)** | **-0.13* (0.06)** |
| Binary (Good/Very Good/Excellent vs. Poor/Fair) | **-0.02* (0.01)** | -0.01 (0.01) |

Notes: Standard errors are in parentheses and clustered by the respondent's region. Analysis of NHIS data 2019-2023 using Ordinary Least Square (OLS) models. Estimates are weighted by NHIS sampling weights and adjusted for the following demographic factors - age, sex, race, education, marital status, citizenship, language, region of residence, labor force participation, poverty status, years in the US, and year and region fixed effects. Sample size for the 2021-2023 analysis ranges from 9,954 to 9,983. * p < 0.10, ** p < 0.05, *** p < 0.01. NHIS: National Health Interview Survey. S.E.: Standard error.

**Supplemental Table 2:** Differences in health insurance, access to care, healthcare utilization, and health status between rural and urban non-US-born 65 and above Immigrants, 2019-2023 NHIS.

| **Outcome Variables** | **Coefficient (S.E.)**  **18-64-year-olds** | **Coefficient (S.E.)**  **65+-year-olds** |
| --- | --- | --- |
| **Health Insurance** | |  |
| Any health insurance coverage | -0.07 (0.04) | -0.04 (0.04) |
| Private health insurance coverage | **-0.04* (0.01)** | **0.06* (0.02)** |
| Health insurance through Medicaid | -0.03 (0.02) | -0.02 (0.03) |
| **Access to Care** | |  |
| Has usual place for medical care | **0.03* (0.01)** | -0.00 (0.01) |
| Problems paying or unable to pay medical bills, past 12 months | **0.03** (0.01)** | 0.03 (0.03) |
| Delayed filling prescription to save money, past 12 months | **0.02** (0.00)** | **-0.02* (0.01)** |
| **Healthcare Utilization** | |  |
| Was in a hospital overnight in past 12 months | 0.01 (0.01) | -0.04 (0.03) |
| Number times visited urgent care in past 12 months | **-0.09** (0.02)** | **-0.13* (0.04)** |
| Number times in ER/ED in past 12 months | **0.04* (0.02)** | **-0.11* (0.04)** |
| Last doctor visit was a wellness visit | -0.02 (0.03) | 0.03 (0.02) |
| **Self-Reported Health Status** | |  |
| Five Categories (5=Excellent, 1=Poor) | **-0.11** (0.02)** | -0.02 (0.07) |
| Binary (Good/Very Good/Excellent vs. Poor/Fair) | **-0.02* (0.01)** | -0.06 (0.03) |

Notes: Standard errors are in parentheses and clustered by the respondent's region. Analysis of NHIS data 2019-2023 using Ordinary Least Square (OLS) models. Estimates are weighted by NHIS sampling weights and adjusted for the following demographic factors - age, sex, race, education, marital status, citizenship, language, region of residence, labor force participation, poverty status, years in the US, and year and region fixed effects. Sample size ranges from 4,768 to 4,780. * p < 0.10, ** p < 0.05, *** p < 0.01. NHIS: National Health Interview Survey. S.E.: Standard error.
